# Use of a mutation-specific genotyping method to assess for HIV-1 drug resistance in antiretroviral-naïve HIV-1 Subtype C-infected patients in Botswana

**DOI:** 10.12688/aasopenres.13107.2

**Published:** 2021-05-07

**Authors:** Dorcas Maruapula, Iain J. MacLeod, Sikhulile Moyo, Rosemary Musonda, Kaelo Seatla, Kesaobaka Molebatsi, Melvin Leteane, Max Essex, Simani Gaseitsiwe, Christopher F. Rowley

**Affiliations:** 1 Botswana Harvard AIDS Institute Partnership, Gaborone, Botswana; 2 University of Botswana, Gaborone, Botswana; 3 Harvard T.H Chan School of Public Health, Boston, MA, USA; 4 Aldatu Biosciences, Watertown, MA, USA; 5 Beth Israel Deaconess Medical Center, Boston, MA, USA

**Keywords:** HIV-1 drug resistance testing, Assay performance, Pan-degenerate amplification and adaptation

## Abstract

**Background:** HIV-1 drug resistance poses a major threat to the success of antiretroviral therapy. The high costs of available HIV drug resistance assays prohibit their routine usage in resource-limited settings. Pan-degenerate amplification and adaptation (PANDAA), a focused genotyping approach based on quantitative PCR (qPCR), promises a fast and cost-effective way to detect HIV drug resistance mutations (HIVDRMs).  Given the high cost of current genotyping methods, we sought to use PANDAA for screening key HIVDRMs in antiretroviral-naïve individuals at codons 103, 106 and 184 of the HIV-1 reverse transcriptase gene. Mutations selected at these positions have been shown to be the most common driver mutations in treatment failure.

**Methods:** A total of 103 samples from antiretroviral-naïve individuals previously genotyped by Sanger population sequencing were used to assess and verify the performance of PANDAA. PANDAA samples were run on the ABI 7500 Sequence Detection System to genotype the K103N, V106M and M184V HIVDRMs. In addition, the cost per sample and reaction times were compared.

**Results:** Sanger population sequencing and PANDAA detected K103N mutation in three (2.9%) out of 103 participants.  There was no evidence of baseline V106M and M184V mutations observed in our study. To genotype the six HIVDRMs it costs approximately 40 USD using PANDAA, while the reagents cost per test for Sanger population sequencing is approximately 100 USD per sample. PANDAA was performed quicker compared to Sanger sequencing, 2 hours for PANDAA versus 15 hours for Sanger sequencing.

**Conclusion:** The performance of PANDAA and Sanger population sequencing demonstrated complete concordance. PANDAA could improve patient management by providing quick and relatively cheap access to drug-resistance information.

## Introduction

HIV remains a major global health problem; currently, 37.9 million adults and children are estimated to be living with HIV with sub-Saharan Africa being the most severely affected region ^1^. In Botswana, 380 000 people are estimated to be living with HIV of which 310 713 are on treatment ^2^. In 2016, Botswana introduced universal HIV treatment to all HIV positive individuals regardless of their immune status ^3^. Combination antiretroviral therapy (cART) has been successful in reducing morbidity and mortality in individuals infected with HIV as well as in prevention of mother-to-child transmission (PMTCT) of HIV ^4^. Despite the availability of antiretroviral drugs, which inhibits HIV replication and reducing mortality, one public health concern about the wide scale rollout of cART is the increase in emergence and transmission of HIV drug resistance ^5– 7^, which has the potential to reduce the efficacy and compromise the success of ART programmes ^8, 9^.

Although first generation NNRTIs, Nevirapine(NVP) and Efavirenz(EFV) have been replaced by DTG as part of the first line cART regimen, presence of baseline NNRTI resistance mutations has been linked to poor response to first line DTG based regimen ^10^, therefore it is still important to analyze NNRTI mutations that would affect the efficacy of DTG based regimen. HIV-1 reverse transcriptase, protease and integrase mutations introduced into the viral genome contribute to the development of resistance to antiretroviral drugs. Major non-nucleoside reverse transcriptase inhibitor (NNRTI) mutations, such as K103N and V106M, are selected when HIV is exposed to nevirapine (NVP) and efavirenz, which is still used in both low and high resource settings as part of patient management. Also, resistance mutations that develop in patients exposed to the first generation NNRTIs, NVP and EFV have been shown to confer some cross-resistance to second generation NNRTIs like etravirine and rilpivirine ^11^. M184V is a major NRTI mutation selected for under tenofovir and lamivudine. HIV drug resistance testing is routinely used for clinical care in high-income countries; however, routine HIV drug resistance testing is not available to majority of patients in resource-limited settings due to the high costs of implementation and limited trained manpower. While Sanger sequencing-based methodologies remain the gold standard for mutation detection, the assays are costly and resource-intensive. Thus, it is urgent to use a simple and cheaper detection method for HIV drug resistance. Detecting known specific mutations provides important information that guides patient treatment options. Moreover, utilising point mutation assays could provide a faster crucial information regarding the mutations present in the patient.

In this study, we compare an HIV genotyping method, pan-degenerate amplification and adaptation (PANDAA), a focused point mutation genotyping assay, with Sanger population based sequencing ^12^. It is anticipated that PANDAA could serve as an alternative method to rapidly detect HIV-1 drug resistance mutations in HIV patients in Botswana.

## Methods

### Study population

This was a retrospective study utilizing existing data and stored PCR products from 103 specimens previously genotyped by Sanger based population sequencing from a previous completed study conducted at Botswana Harvard AIDS Institute Partnership, Gaborone, Botswana: Novel strategy for HIV drug resistance monitoring in developing countries (BHP063 study) ^13^. Briefly, this study enrolled 234 pregnant women diagnosed with HIV and 188 pre-ART from Infectious Diseases Care Clinics (IDCC) between 2012 and 2015. These participants were enrolled to determine the prevalence of HIV transmission at three different geographical locations in Botswana (Gaborone, Molepolole, Mochudi). In samples collected between 2014 and 2015, the following mutations were detected in the main cohort; K103N, G190A and L90M ^13^.

 For the current study, a convenience sampling method was employed to maximize the number of samples available for analysis and the current study used baseline samples collected between 2014 and 2015 from the main cohort, provided that the stored sample(s) were still available with sufficient remaining volume for PCR products. At the time of the current study, the first-line ART regimen in Botswana consisted of tenofovir + emtricitabine (or lamivudine) + efavirenz (or NVP).

### Ethical considerations

Ethical clearance for the BHP063 study was obtained from the Human Research Development Committee (HRDC) at the Botswana Ministry of Health (Ethics permit number: HPDME 13/18/1 Vol (366). All participants consented prior to participation in the study.

The current study was approved by the University of Botswana Institute Review Board (IRB) and the Human Research Development Committee at the Botswana Ministry of Health (Ethics permit number: HPDME 13/18/1 Vol (833)) and the need for informed consent was waived since remnant plasma samples were used for this study.

### RNA extraction, reverse transcription and PCR amplification

RNA extraction using EZ1 Advanced XL (Qiagen) automated instrument and PCR were performed in the main cohort as described previously ^13^. The primers used were CWF1-LNA2 and CWR1-LNA3 for first round, whereas second-round primers were CWF1-LNA2 and RT20C13 ( Table 1).

**Table 1.** Detailed sequences of the primers used for PCR and sequencing ^13^.

Primer Name	Primer Sequence	HXB2 position
CWF1-LNA2	5′+GAA+G+GACACCAAATGAAAGAYTG-3′	2044-2066
CWR1-LNA3	5′-G+CA+TAC+TTYCCTGTTTTCAG-3′	3613-3594
**CWF1**	5′ **-GAAGGACACCAAATGAAAGAYTG-**3′	**2044-2066**
**CWCS2**	5′ **-AGAACTCAAGA CTTTTGGG-**3′	**2044-2066**
**CWCS3**	5′ **-TGCTGGGTGCGGTATTC-**3′	**3145-3129**
**CWCS5**	5′ **-TGGTAAA TTTGATATGTCCAT-**3′	**3577-3557**
**Seq6**	5′ **-CCATCCCTGTGGAAGCACATTA-**3′	**3008-2987**
**Seq2.1-F2**	5′ **-GGCCAGGGAATTTTCTTCAGAGC-**3′	**2120-2142**
**RT20C**	5′ **-CTGCCAATTCTAATTCTG CTTC-**3′	**3462-3441**

The primers used for Sanger sequencing are those shown in bold.

### Drug resistance genotyping by population sequencing

Direct population sequencing of the pol gene was previously performed on an ABI 3130xl genetic analyser (Applied Biosystems, Foster City, CA, USA) using BigDye Terminator cycle sequencing kit (Life Technologies, Carlsbad, CA, USA) ^13^. 

### PANDAA qPCR

The stored pol-derived PCR products were diluted prior to PANDAA focused genotyping.

PANDAA qPCR reactions for detecting drug-resistant point mutations K103N, M184V, V106M were performed on an ABI 7500 real-time PCR System (Applied Biosystems).

**Figure 1.**
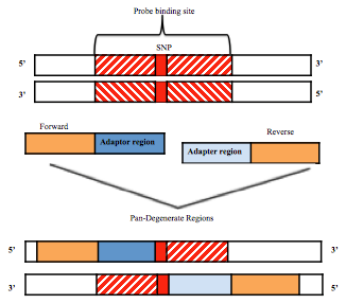
Overview of PANDAA. Adaptor regions of PANDAA primers that is matched to the probe-binding site and a pan-degenerate region. This figure has been reproduced with permission from MacLeod *et al*. ^12^.

PANDAA is provided as a 10x mix of primers and probes that are specific for each DRM in three triplex qPCR reactions ^14^. A single target codon is amplified by the PANDAA primers (proprietary properties of Aldatu Biosciences) and the wild-type variants in each patient is detected using a VIC-labelled TaqMan MGB probe, which is differentiated from the resistant variant, which is detected by a FAM-labelled probe (Life Technologies, MA, USA). Components of the PANDAA reaction contained 5 µL buffer (kappa Probe Fast, kappa Biosystems), 1 µL PANDAA probes (VIC labelled wild-type and DRM-specific FAM-labelled) and primer mix (forward and reverse primers), 4 µL template to a final volume of 10 µl. Each sample was performed in triplicate under the following thermal cycling conditions: 98°C for 3 minutes followed by 40 cycles of 95°C for 5 seconds then 60°C for 90 seconds during which fluorescence data were acquired. Each sample was run in triplicate for each DRM. PANDAA primers include locked nucleic acids (LNAs) which increase affinity for their target sequences and contain an adaptor region (ADR) that is matched to the probe-binding site and a pan-degenerate region (PDR) that incorporates degenerate bases in the targeted primer-binding site upstream of the ADR. The principle of PANDAA is shown in Figure 1 ^12^.

The different protocols (K103N, V106M and M184V) were performed separately, each with a corresponding set of standards.

### PANDAA data analysis

The threshold was set at 0.02 and using the ABI 7500 software, raw qPCR fluorescence data were exported from Applied Biosystems SDS software to excel and Cq values were corrected for differences in probe-binding efficiencies. All reactions were performed in triplicate, and the mean of the three values was used for calculation.

Cycle quantification (Cq) values were recorded for each sample. Samples were considered positive when the amplification of the mutant was statistically significant with respect to control sample. The percent abundance of the DRM was calculated using E^ΔCq, whereby E is the efficiency of probe-binding, and ΔCq is the Cq difference between the wild-type and DRM probes, after correcting for variations in probe-binding efficiency.

### Reagent cost comparison

The costs of reagents were estimated according to updated prices. Cost of equipment such as ABI 3130XL and ABI 7500 real-time PCR system were not considered as these items of equipment were already available in the laboratory.

### Reaction time

To establish the total time to perform each method, we considered the total time to perform genotyping method and interpretation of results.

### Concordance statistics

Agreements between PANDAA and Sanger population sequencing were calculated using Cohen’s kappa coefficient. The Mann-Whitney U-test was used to test for differences in CD4 counts and viral loads between the groups with drug resistance mutations and those without drug resistance mutations. Two-sided tests were used and a p-value less than 0.05 implied statistically significant differences. All statistical analysis was carried out using R version 3.5.1 ^15^, other than R ^2^, which was calculated using the linear regression function in Microsoft Excel.

## Results

### Characteristics of participants

All participants were female. The median age was 28 (Q1; Q3: 24; 32) years ( Table 2).

**Table 2.** Characteristics of participants.

Characteristics	Value
Age, median (Q1, Q3) years	28 (24, 32)
CD4+ T cell count, median (Q1,Q3) (cells/uL)	331 (207.5, 495.5)
HIV-1 RNA copies, median (Q1, Q3), log _10_ copies/ml (Q1,Q3)	4.1 (3.49, 4.55)

### Performance of PANDAA

**Figure 2.**
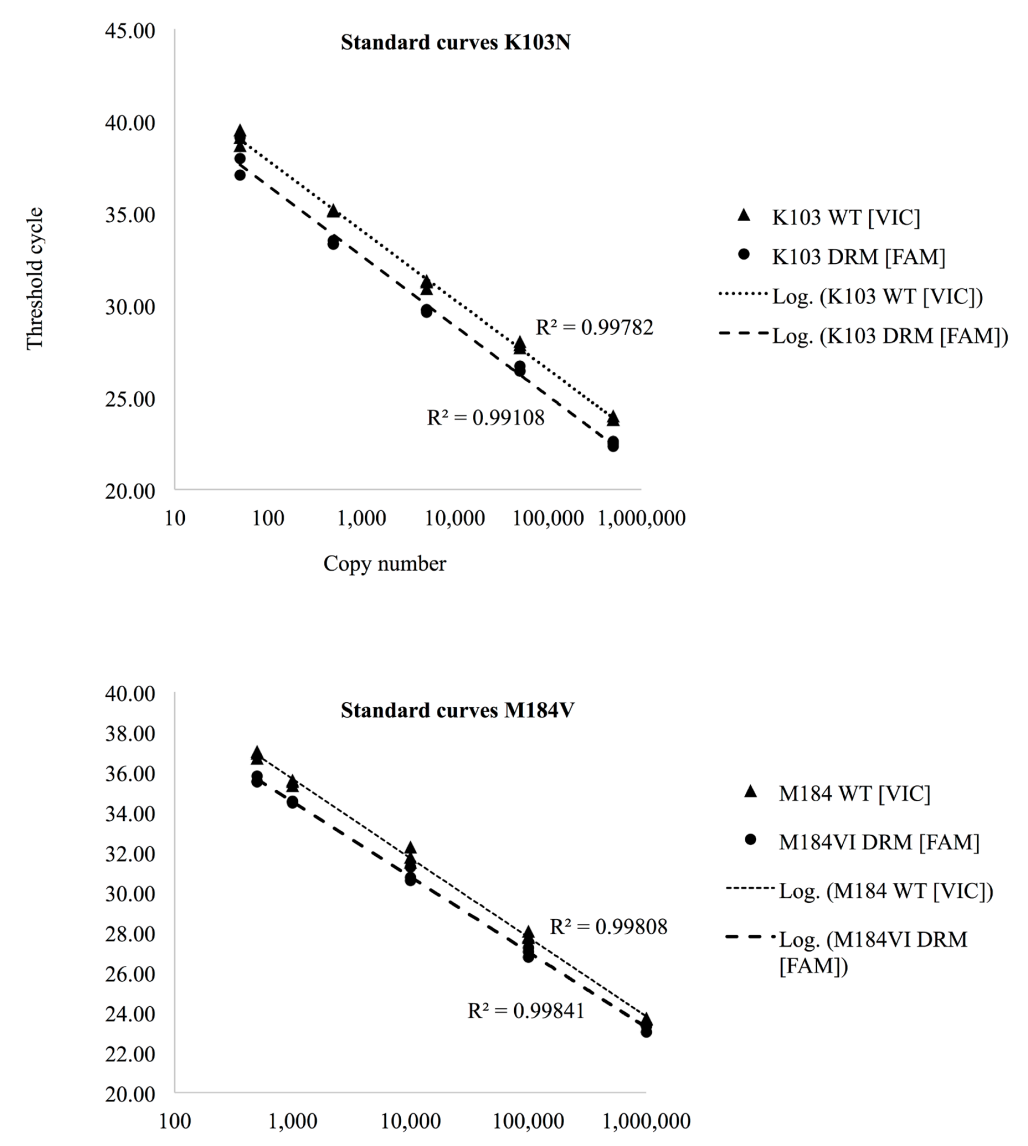
Standard curves generated from ten-fold serial dilutions. Correlation coefficients (r ^2^) were higher than 99.4%.

The amplification efficiency was determined by analysing serial dilutions of positive control. A linear standard curve generated from 10-fold dilution was obtained as shown in Figure 2.

**Figure 3.**
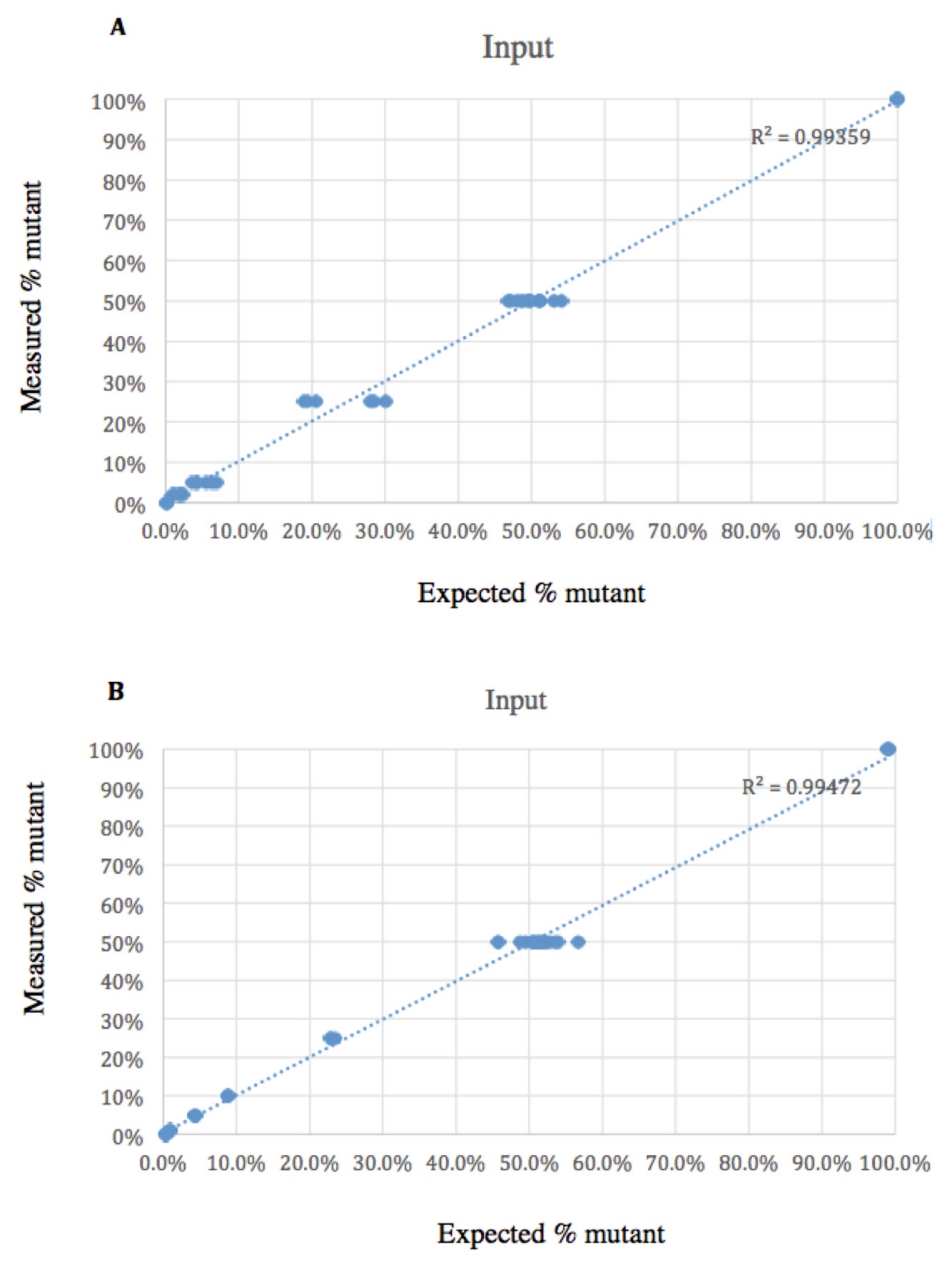
Measured mutant correlated with expected mutant. ( **A**) K103N: R ^2^=0.99339. ( **B**) M184V: R ^2^=0.99472.

PANDAA showed reproducible results when 1:1 mix of wild-type and DRM templates over a range of copy numbers tested in triplicate. The correlation of each mutant detected by PANDAA correlated with expected mutant as shown in Figure 3.

### Quantification of drug resistance of patient samples by PANDAA

PANDAA was completed on patient-derived amplicons of 103 ARV naïve individuals for the K103N, V106M and M184V DRMs using PANDAA. PANDAA identified the presence of K103N in three samples. The three samples with K103N were the same samples that Sanger sequencing detected. Only wild-type sequences at codons 106 and 184 of the RT could be identified by both PANDAA and population sequencing. There was a complete concordance between population sequencing and PANDAA assay as PANDAA qPCR confirmed the presence of HIV drug-resistant mutations as identified by population-based sequencing as shown in Table 3.

**Figure 4.**
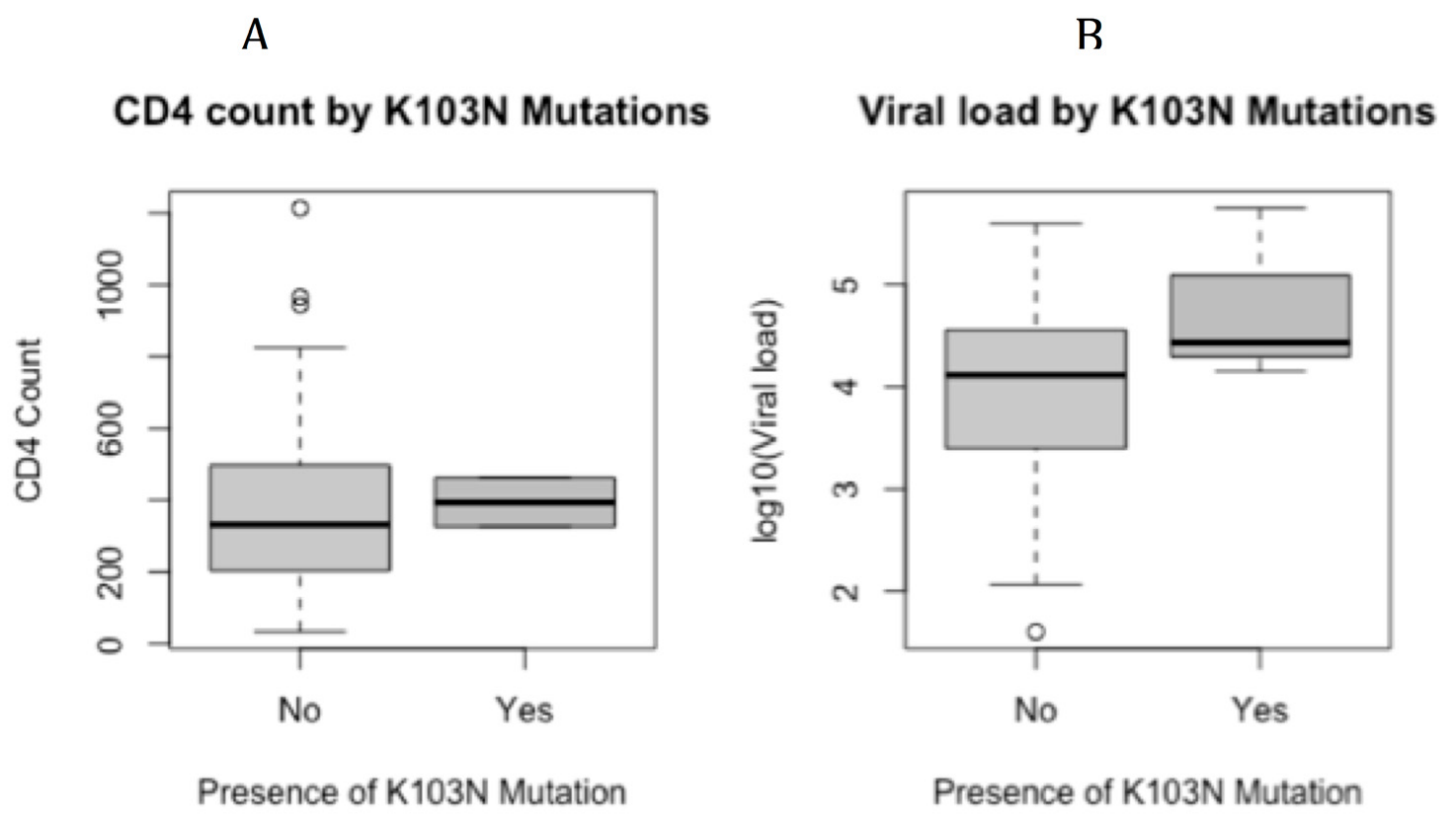
Median CD4 ( **A**) and median viral load ( **B**) between samples with detected K103N and samples without K103N mutation. Samples without K103N mutation (n=100). Samples with K103N mutation (n=3)

Differences in CD4 counts and viral loads between the groups with drug resistance mutations and those without drug resistance mutations are shown in Figure 4A and Figure 4B.

**Table 3.** Comparison of drug resistance mutations identified by Sanger sequencing and PANDAA.

			PANDAA		
	Assay		Yes	No	Total
**K103N**	**Sanger**	**Yes**	3	0	3
		**No**	0	100	100
**M184V**	**Sanger**	**Yes**	0	0	0
		**No**	0	103	103
**V106M**	**Sanger**	**Yes**	0	0	0
		**No**	0	103	103

### Cost and time analysis of each reaction

We calculated the costs for materials and reagents including those associated with the running of samples on the ABI 3130XL sequencer.

The cost of genotyping six drug resistance mutations per patient using PANDAA is 40 USD and Sanger population sequencing is estimated at 100 USD per sample. The turnaround time for PANDAA and Sanger sequencing is approximately 2 hours and 24 hours, respectively ( Table 4).

**Table 4.** Comparison of sequencing cost and time required for PANDAA and Sanger population sequencing.

Sequencing method	Laboratory parameter	Time (Minutes)		Cost /sample US$
		Estimated Hands on time	Instrument time	
Sanger Sequencing **	RNA extraction	20mins	43min	12
	RT-PCR	10mins	240min	10.68
	Nested PCR	10mins	180min	2
	Gel electrophoresis	10mins	30min	4.45
	PCR product purification	20mins	-	1.45
	Cycle sequencing	10mins	106mins	20.5
	Sequence purification	15mins	-	3.00
	Sequence detection	10mins	420mins (Overnight)	
	Data analysis	20mins		
	**Total**	**1hr 20mins**	**19hrs 12min**	**$54.08**
PANDAA *	RNA extraction	10min	86min	12
	One-step RT-PCR	10minutes	120min	24
	Data analysis	20min	20min	
	**Total**	**40min**	**4hrs 16mins**	**$36**

These cost do not include costs for gloves, tips and instruments. ** Batch of 13 samples and seven primers *Batch of 32 samples in triplicate.

## Discussion

Here, we show that the HIV drug resistance mutations results of PANDAA are comparable to those produced by Sanger population sequencing. Our study provides baseline data of PANDAA performance and has added an insight that monitoring HIV drug resistance mutations is possible using PANDAA. Having protocols in place for detecting HIV drug resistance mutations using fast and low-cost platforms is important for guiding treatment options and patient management, thereby achieving WHO goal of eliminating HIV by 2030.

When the duration of each method was compared, the results showed that PANDAA required the shortest time for genotyping and had the lowest cost, when compared to Sanger sequencing. It is important to note that PANDAA cost 40 USD for six relevant drug resistance mutations, thus making it much more affordable compared to Sanger sequencing which costs. Sanger sequencing is the widely used and validated method and it is still a relevant platform to use; however, using PANDAA to detect key drug resistance mutations will reduce the cost, especially in this test-and-treat era, thereby enabling quicker results to patients. 

Our study had small number of positive samples used to compare the results; however, PANDAA was shown to produce concordant results with sanger sequencing. PANDAA can be considered to rapidly detect drug resistance mutations at a cheaper cost. In addition, PANDAA kit is more cost-effective, and after preparation genotyping results can be obtained in less than two hours.

Botswana has recently introduced universal HIV therapy; however, additional patients are likely to develop drug resistance and transmit these drug-resistant HIV strains to their uninfected partners. As more patients will be receiving ART in Botswana, there is a need to consider investing in fast, low-cost assays to detect mutations associated with drug resistance.

Although most patients are currently initiating on DTG based regimen in Botswana, efavirenz-based regimen is still being used for pregnant women ^16, 17^ and patients on TB treatment ^18^. Common drug resistance mutations associated with resistance to efavirenz include K103N (AAA/G to AAC/T) and V106M ^19^. The key M184V (ATG to GTG) mutation in HIV-1 RT is associated with high-level resistance to the lamivudine (3TC) and emtricitabine (FTC) ^20^; however, M184V has been shown to rapidly decay in the absence of treatment as a result of its impact on viral fitness ^21^. HIVDR testing is important to clinicians for patient management, however the cost of reagents and equipment maintenance for resistance testing is the biggest obstacles in resource-limited settings.

In this study, we used PANDAA, to screen for NRTI and NNRTI drug-resistant viruses in 103 newly diagnosed HIV-infected pregnant women from the BHP063 cohort and compared the PANDAA results to those obtained by Sanger based population sequencing. Standard curves generated proved PANDAA to accurately differentiated mutants from wild type. In one hundred and three samples included in our study, the use of PANDAA assay enabled detection of K103N in 3 antiretroviral naïve individuals. Both PANDAA and Sanger sequencing did not detect any mutations at codons 106 and 184 in the HIV strains from this cohort. This study provides insights on the performance of PANDAA, a simple method that utilises primers and probes on any available real-time qPCR platform to detect key HIV drug resistance mutations.

The data generated by our study confirm the ability of PANDAA to detect K103N HIV drug resistance mutation as a point mutation assay, and these data correspond to Sanger sequencing data. The results generated from the use of PANDAA provide evidence that this assay represents an alternative strategy for rapid, specific detection of mutations of interest. At the time the samples were collected for this study, 2014–2015, the standard of care for treatment of HIV infection in Botswana was a regimen that included tenofovir, emtricitabine, and efavirenz co-formulated into one pill, Atripla, taken once a day. By using PANDAA, we targeted the most likely mutations to develop to these medications in HIV-1 subtype C, the M184V, K103N and V106M mutations in reverse transcriptase, a targeted and cost-effective approach to genotyping is possible ^22^.

Our study had some limitations. Firstly, we only examined the most common relevant resistant mutations, V106M, K103N and M184V of the reverse transcriptase; therefore, there was a limited number of positive mutations available for analysis. There was no clear correlation between viral load and mutations identified due to small sample size of patients with mutations. Secondly, at the time of the study we utilized samples from ART naïve patients and not exposed to ART leading to few cases with drug resistant mutations. Another limitation in this study was the lack of samples with M184V and V106M, making it difficult to draw a conclusion on the performance of PANDAA in detecting V106M and M184V. There is a need for further studies utilising samples with more HIV drug mutations. The applicability of this assay can be demonstrated further by testing a larger number of samples with known mutations. Nevertheless, we have shown that it is possible to genotype HIV drug resistance mutations in HIV naïve individuals using PANDAA and future work will build on the findings of this study.

## Conclusion

Our findings proved the potential use of PANDAA assay for testing drug resistance mutations in resource-limited settings. This study demonstrates that applying this cost-effective assay to samples from treatment-naïve individuals where background HIV drug resistance may be increasing can provide valuable insight into baseline resistance and allow for decisions to be made to ensure the best prospect of successful HIV treatment. PANDAA holds the same promise for detection of HIV DRM in patients failing ART, although the current study did not include any participants with known ART exposure. Given the simplicity and cost-effectiveness of PANDAA, it can be performed in any laboratory with real-time PCR capability and its principle could be easily adapted to other clinically relevant point mutations. Overall, the comparative results indicate that PANDAA assay provides similar results with Sanger population sequencing at a much lower cost.

## Data availability

Sequence data generated in this study has been deposited with NCBI GenBank under sequential accession numbers MT908833– MT908846 and MT919428– MT919516.

Figshare: Use of a mutation-specific genotyping method to assess for HIV-1 drug resistance in antiretroviral-naïve HIV-1 Subtype C-infected patients in Botswana; https://doi.org/10.6084/m9.figshare.12644930 ^23^.

Data are available under the terms of the Creative Commons Attribution 4.0 International license (CC-BY 4.0).
