# Supplementary material for: Use of a mutation-specific genotyping method to assess for HIV-1 drug resistance in antiretroviral-naïve HIV-1 Subtype C-infected patients in Botswana
Source: AAS Open Res. 2021 May 7;3:50. Originally published 2020 Sep 28. [Version 2] doi: 10.12688/aasopenres.13107.2 (PMC8112461; doi:10.12688/aasopenres.13107.2)
Supplement: Peer review history

# Peer review: Reviewer response for version 2

**DOI:** 10.21956/aasopenres.14334.r28573

**Published:** 2021-05-10

**Authors:** Justen Manasa, Vinie Kouamou

**Affiliations:** 1 Department of Medical Microbiology, College of Health Sciences, Univeristy of Zimbabwe, Harare, Zimbabwe; 2 Department of Medicine, College of Health Sciences, University of Zimbabwe, Harare, Zimbabwe

The authors have addressed our previous comments adequately.

Is the work clearly and accurately presented and does it cite the current literature?

Partly

If applicable, is the statistical analysis and its interpretation appropriate?

Yes

Are all the source data underlying the results available to ensure full reproducibility?

Yes

Is the study design appropriate and is the work technically sound?

Partly

Are the conclusions drawn adequately supported by the results?

Partly

Are sufficient details of methods and analysis provided to allow replication by others?

Partly

Reviewer Expertise:

Molecular Virology, Bioinformatics, Molecular Diagnostics, HIV

We confirm that we have read this submission and believe that we have an appropriate level of expertise to confirm that it is of an acceptable scientific standard.

# Peer review: Reviewer response for version 1

**DOI:** 10.21956/aasopenres.14207.r28046

**Published:** 2020-11-03

**Authors:** Diana B. Dickinson

**Affiliations:** 1 Independence Surgery, Gaborone, Botswana

This is a very relevant, clearly presented and well done paper, readily reproducible, particularly in the context of a country, Botswana, that has very strong guidelines such that there is a standard universal first line regimen for both public and private facilities. This study shows us that a cost effective and rapid way is possible to demonstrate any pre-existing drug resistant mutations in all patients pre-initiation of treatment. We would have liked to have been doing that for years, but the cost of universal genotyping using Sanger sequencing was too much for us to do routinely. 

 Fortunately, as this study shows, our level of transmitted drug resistance in the untreated population is low, however with the PANDAA assay as demonstrated by this study, routine screening for important mutations would be possible, still keeping to a budget, enabling rapid response if relevant mutations are detected.

 Of course, now our first line has changed to an integrase inhibitor regimen, the exact point mutations would have to be changed appropriately, but as the investigators point out this is easily done.

 It would also be useful to use this modified PANDAA in the rare cases of treatment failure expected, to give a rapid overview on important mutations to craft a salvage regimen while awaiting the full Sanger sequence to fine tune that regimen.

 I hope that this study can be used for our national guidelines to focus more easily and effectively on specific mutations expected to be revealed by our local PANDAA modifications.

Is the work clearly and accurately presented and does it cite the current literature?

Yes

If applicable, is the statistical analysis and its interpretation appropriate?

Yes

Are all the source data underlying the results available to ensure full reproducibility?

Yes

Is the study design appropriate and is the work technically sound?

Yes

Are the conclusions drawn adequately supported by the results?

Yes

Are sufficient details of methods and analysis provided to allow replication by others?

Yes

Reviewer Expertise:

Clinical HIV specialist witha specific interest  in resistance and complicated or failing patients

I confirm that I have read this submission and believe that I have an appropriate level of expertise to confirm that it is of an acceptable scientific standard.

# Peer review: Reviewer response for version 1

**DOI:** 10.21956/aasopenres.14207.r27974

**Published:** 2020-10-14

**Authors:** Justen Manasa, Vinie Kouamou

**Affiliations:** 1 Department of Medical Microbiology, College of Health Sciences, Univeristy of Zimbabwe, Harare, Zimbabwe; 2 Department of Medicine, College of Health Sciences, University of Zimbabwe, Harare, Zimbabwe

**Abstract:**

 Background:

1. The combination of mutations evaluated in this study is suboptimal in the context of the current ISTI based regimens. In the context of a TLD regimen, a panel of mutations that includes at least the K65R mutation for TDF in addition to the M184V mutation in the current panel would be much more useful.
2. The statement that, "Mutations selected at these positions have been shown to be the most common driver mutations in treatment failure" is no longer true in the context of the current regimens. This is a historical statement and it should be clearly stated. This will allow the evaluation of mutations that are important to the current regimens.

Methods:

1. The ABI 7500 system is not a sequence detection system BUT a real time PCR system.

Results:

1. The costing comparison is between detecting six mutations using PANDAA vs Sanger sequencing. However, the study evaluated the detection of only 3 mutations?
2. The time comparison only focuses on the real time PCR vs sequencing, it would be more objective to add the first half of the work, RNA extraction to PCR.

 

**Introduction:**

1. "HIV-1 reverse transcriptase mutations introduced into the viral genome contributes to the development of resistance to antiretroviral drugs" - Mutations in the PR, Integrase genes also contribute to ART resistance.
2. The statement that, "Major non-nucleoside reverse transcriptase inhibitor (NNRTI) mutations, such as K103N and V106M, are selected when exposed to nevirapine (NVP), which is still used in low resource settings as part of management." omits the role of EFV in the selection of those two mutations and EFV is also used in RLS. It’s the drug that was in use at the time of collection of the samples used in this study.
3. "Studies have shown that >98% of patients failing first-line NNRTI regimens would have one or more drug resistance mutations, such as K103N, V106M and M184V" is highly inaccurate. I believe the statement originated from Rhee *et al. *(2015 ^1^) which implied that the set six mutations (K65R, M184V, K103N, V016M, Y181C, and G190A) was 98% sensitive in detecting HIVDR in treatment failure in RLS at that time, taking into consideration the regimens that were is use. Furthermore, if one mentions "studies" it is expected that they provide multiple references or a single systematic review as the reference.
4. "Moreover, utilising point mutation assays reduces the need for several laboratory equipment". This statement may be misleading, especially in reference to the PANDAA assay. The major difference between PANDAA and Sequencing is the method used in the detection of the mutations. One uses real-PCR to detect specific point mutations and the other uses sequencing. The extraction, reverse transcription and PCR are similar. When doing such comparisons, one should be cognoscente of the fact that there are now low cost sequencing platforms that may be even cheaper that some of the real time PCR platforms on the market.

 

**Methods:**

1. It's not clear which other samples, other than the Gaborone ANC 2014-2015 (K103N, n=4) samples were used for this study. Please clarify this. This is key to understanding the analysis that was done, the results and their interpretation.
2. The investigators used amplicons that were generated during the sequencing process for the PANDAA evaluation. If this is correct, there is no need to provide details of RNA extraction, reverse transcription, PCR and PCR product clean up. This would have been reported already in the primary paper.
3. When reporting target regions for primers (Table 1), its important to indicate the reference, for example HXB2 if that is the reference you used.
4. Did the investigators do any sequencing for this paper, if not, there is no need to provide details of the method. Reference should be made to the primary paper.
5. "All reactions were performed in triplicate, and the mean of the three values was used for calculation", isn't it better to use median rather than mean?
6. For cost comparison, the investigators should indicate the cost inputs.
7. The analysis of the differences in CD4 counts and viral loads between the groups with drug resistance and those without, seems to be out of place. It's not indicated anyway in the objectives of the study. Nothing about this is even mentioned in the introduction. The power of this analysis is further diminished by the small number of samples with HIVDR mutations (3) in this study.

 

**Results:**

1. The investigators mention that they did an analysis to determine the "lower limit of detection" for PANDAA but the result is not indicated.
2. Turnaround time (Table 4).  For the statement that the turn around time for PANDAA is 2 hrs and that of Sanger is 24 hrs to be objective, one needs to highlight that this time is just for the real time PCR vs Sequencing, excluding RNA extraction, Reverse transcription, PCR, and PCR clean up. They should also provide the sequencing protocol as well as the sequencing clean up protocol. In general the sequencing PCR should not take more that 1.5 hrs, the clean up 30 minutes and the sequencing electrophoresis, not more than 1.5 hours per injection. If you have a 16 capillary array, your turn around time will be much shorter compared to the 8 capillary array. So, its important to indicate all this information so as to determine how the 15 hrs came about.

 

**Discussion:**

1. This evaluation or comparison would have benefited more from a sample with higher proportions of the different mutations evaluated. 
2. The WHO 90-90-90 goals target the elimination of HIV as a public health threat by 2030 NOT 2020.
3. Turn around times need to be assessed in the context of the workflow in patient management. For example, if a patient has to go home and come and collect results after 48hrs, a difference of 13 hrs will not add value for the patient. However, if PANDAA enabled same day delivery of results, then, it would make a significant difference in patient management. Unfortunately the authors only focused on the time post PCR, which gives an illusion that it takes 2 hrs to get results for PANDAA. A proper assessment would have to be from RNA extraction to genotyping result. 
4. The statement that, in Botswana, NVP is still being used as prophylaxis for prevention of mother to child transmission of HIV, is mostly likely in accurate.  Please provide a reference like the current HIV management guidelines for Botswana.
5. The authors properly highlight the limitations of this study, some which have been highlighted above. However these are very strong limitations which will significantly reduce the impact of this paper. 

Is the work clearly and accurately presented and does it cite the current literature?

Partly

If applicable, is the statistical analysis and its interpretation appropriate?

Yes

Are all the source data underlying the results available to ensure full reproducibility?

Yes

Is the study design appropriate and is the work technically sound?

Partly

Are the conclusions drawn adequately supported by the results?

Partly

Are sufficient details of methods and analysis provided to allow replication by others?

Partly

Reviewer Expertise:

Molecular Virology, Bioinformatics, Molecular Diagnostics, HIV

We confirm that we have read this submission and believe that we have an appropriate level of expertise to state that we do not consider it to be of an acceptable scientific standard, for reasons outlined above.

## References

[1] : HIV-1 Drug Resistance Mutations: Potential Applications for Point-of-Care Genotypic Resistance Testing. *PLoS One*.2015;10(12) : 10.1371/journal.pone.0145772 e0145772 10.1371/journal.pone.0145772 26717411PMC4696791

# Response

**Published:** 2021-04-13

**Authors:** Dorcas Maruapula

**Affiliations:** Botswana Harvard AIDS Partnership, Botswana

**Authors’ Response to Reviewers’ Comments:**

 Manuscript Title: Use of a mutation-specific genotyping method to assess for HIV-1 drug resistance in antiretroviral-naïve HIV-1 Subtype C-infected patients in Botswana

 We thank the reviewers for their valuable comments, suggestions and insights that have helped us improve our manuscript. We have revised the manuscript by clearly discussing the limitations of the method in the Discussion section. We hope the reviewers will find our revised manuscript improved and suitable for publication.

 Please find below a point-by-point response to the reviewers comments.

 Sincerely,

 D.M

**Abstract:**

*Background:*

***Reviewer’s comments**; The combination of mutations evaluated in this study is suboptimal in the context of the current ISTI based regimens. In the context of a TLD regimen, a panel of mutations that includes at least the K65R mutation for TDF in addition to the M184V mutation in the current panel would be much more useful.*

**Authors’ response**: We thank the reviewer for the suggestions. The mutations used here are important although they are not all inclusive. Future work can focus on other mutations that were not included here. It is most likely that the list of important mutations to be evaluated will always change and more mutations added to the list.

***Reviewer’s comments**: The statement that, "Mutations selected at these positions have been shown to be the most common driver mutations in treatment failure" is no longer true in the context of the current regimens. This is a historical statement and it should be clearly stated. This will allow the evaluation of mutations that are important to the current regimens.*

**Author’s response**:  We are thankful to the reviewers for the suggestions. We have indicated that the mutations selected were some of the most common driver mutations for treatment failure at the time the study was conducted and for NNRTI based ART regimen. We have also included more literature which shows that baseline NNRTI resistance are linked to poor response to first-line dolutegravir , therefore it is still important to look at NNRTI resistance mutations (MJ Siedner et al, 2020).

 Methods:

1. ***Reviewer’s comment**: The ABI 7500 system is not a sequence detection system BUT a real time PCR system. *
2. **Author’s response**:  We thank the reviewer for the observation. We have corrected the wording in the new manuscript.

Results:

1. ***Reviewer’s comment**: The costing comparison is between detecting six mutations using PANDAA vs Sanger sequencing. However, the study evaluated the detection of only 3 mutations?*

**Author’s response**: We have now clearly stated the costing comparison in the revised manuscript.

1. ***Reviewer’s comment**: The time comparison only focuses on the real time PCR vs sequencing, it would be more objective to add the first half of the work, RNA extraction to PCR.*

**Author’s response**: We thank the reviewer for the suggestion. We have added the first half of the work, RNA extraction to PCR into the manuscript.

**Introduction:**

1. ***Reviewer’s comment:*** *"HIV-1 reverse transcriptase mutations introduced into the viral genome contributes to the development of resistance to antiretroviral drugs" - Mutations in the PR, Integrase genes also contribute to ART resistance.*

**Author’s response**: We thank the reviewer for the comment. We have corrected the sentence.

  

1. ***Reviewer’s comment:*** *The statement that, "Major non-nucleoside reverse transcriptase inhibitor (NNRTI) mutations, such as K103N and V106M, are selected when exposed to nevirapine (NVP), which is still used in low resource settings as part of management." omits the role of EFV in the selection of those two mutations and EFV is also used in RLS. It’s the drug that was in use at the time of collection of the samples used in this study.*

**Author’s response**; We thank the reviewer for the observation. We have improved on the wording of the manuscript.  

1. ***Reviewer’s comment:*** *:"Studies have shown that >98% of patients failing first-line NNRTI regimens would have one or more drug resistance mutations, such as K103N, V106M and M184V" is highly inaccurate. I believe the statement originated from Rhee et al. (2015* *^1^* *) which implied that the set six mutations (K65R, M184V, K103N, V016M, Y181C, and G190A) was 98% sensitive in detecting HIVDR in treatment failure in RLS at that time, taking into consideration the regimens that were is use. Furthermore, if one mentions "studies" it is expected that they provide multiple references or a single systematic review as the reference*

**Author’s response**: We thank the reviewer for the suggestions. We have added more references.

  

1. ***Reviewer’s comment*** *: "Moreover, utilising point mutation assays reduces the need for several laboratory equipment". This statement may be misleading, especially in reference to the PANDAA assay. The major difference between PANDAA and Sequencing is the method used in the detection of the mutations. One uses real-PCR to detect specific point mutations and the other uses sequencing. The extraction, reverse transcription and PCR are similar. When doing such comparisons, one should be cognoscente of the fact that there are now low cost sequencing platforms that may be even cheaper that some of the real time PCR platforms on the market.*

**Author’s response: **We thank the reviewer for the comment. We have corrected the wording of the sentence.

**Methods:**

1. ***Reviewer’s comment*** *: It's not clear which other samples, other than the Gaborone ANC 2014-2015 (K103N, n=4) samples were used for this study. Please clarify this. This is key to understanding the analysis that was done, the results and their interpretation.*

**Author’s response: **We thank the reviewer for the comment. We have now clearly stated the samples. 

1. ***Reviewer’s comment*** *: The investigators used amplicons that were generated during the sequencing process for the PANDAA evaluation. If this is correct, there is no need to provide details of RNA extraction, reverse transcription, PCR and PCR product clean up. This would have been reported already in the primary paper.*

**Author’s response: **We thank the reviewer for the comment. We have highlighted in the manuscript that the amplicons generated were reported elsewhere.

  

1. ***Reviewer’s comment*** *: When reporting target regions for primers (Table 1), its important to indicate the reference, for example HXB2 if that is the reference you used.*

**Author’s response: **We thank the reviewer for the comment. We indicated the reference in the revised manuscript.

  

1. ***Reviewer’s comment*** *: Did the investigators do any sequencing for this paper, if not, there is no need to provide details of the method. Reference should be made to the primary paper.*

**Author’s response: **We thank the reviewer for the suggestions. We will update the reference of the primary paper.

  

1. ***Reviewer’s comment*** *: "All reactions were performed in triplicate, and the mean of the three values was used for calculation", isn't it better to use median rather than mean?*

**Author’s response: **We thank the reviewers for the suggestion. To enable comparisons with previous studies, we also report means.

  

1. ***Reviewer’s comment*** *: For cost comparison, the investigators should indicate the cost inputs.*
**Author’s response: **We thank the reviewer for the suggestion. We have clearly indicated the cost inputs for cost comparison.

 

1. ***Reviewer’s comment*** *: The analysis of the differences in CD4 counts and viral loads between the groups with drug resistance and those without, seems to be out of place. It's not indicated anyway in the objectives of the study. Nothing about this is even mentioned in the introduction. The power of this analysis is further diminished by the small number of samples with HIVDR mutations (3) in this study.*

**Author’s response: **We thank the reviewers for the comments. We have included the above point as a limitation in the revised manuscript.

**Results:**

1. ***Reviewer’s comment*** *:The investigators mention that they did an analysis to determine the "lower limit of detection" for PANDAA but the result is not indicated.*

**Author’s response: **We thank the reviewer for the observation. We have corrected the above text in the manuscript.

  

1. ***Reviewer’s comment*** *: Turnaround time (Table 4).  For the statement that the turn around time for PANDAA is 2 hrs and that of Sanger is 24 hrs to be objective, one needs to highlight that this time is just for the real time PCR vs Sequencing, excluding RNA extraction, Reverse transcription, PCR, and PCR clean up. They should also provide the sequencing protocol as well as the sequencing clean up protocol. In general the sequencing PCR should not take more that 1.5 hrs, the clean up 30 minutes and the sequencing electrophoresis, not more than 1.5 hours per injection. If you have a 16 capillary array, your turn around time will be much shorter compared to the 8 capillary array. So, its important to indicate all this information so as to determine how the 15 hrs came about.*

**Author’s response: **We thank the reviewer for the suggestions. We have highlighted the above comment in the manuscript.

**Discussion:**

1. ***Reviewer’s comment*** *: This evaluation or comparison would have benefited more from a sample with higher proportions of the different mutations evaluated. *

**Author’s response: **We completely agree with the reviewers and we have clarified this point in the Discussion as a limitation. We had to work with the samples that we had at the time the study was conducted.


* *

1. ***Reviewer’s comment*** *: The WHO 90-90-90 goals target the elimination of HIV as a public health threat by 2030 NOT 2020.*

**Author’s response: **We agree with reviewer, and we have now modified this sentence accordingly.


* *

1. ***Reviewer’s comment*** *: Turn around times need to be assessed in the context of the workflow in patient management. For example, if a patient has to go home and come and collect results after 48hrs, a difference of 13 hrs will not add value for the patient. However, if PANDAA enabled same day delivery of results, then, it would make a significant difference in patient management. Unfortunately the authors only focused on the time post PCR, which gives an illusion that it takes 2 hrs to get results for PANDAA. A proper assessment would have to be from RNA extraction to genotyping result. *

**Author’s response: **A proper assessment was addressed in the revised manuscript.

1. ***Reviewer’s comment*** *: The statement that, in Botswana, NVP is still being used as prophylaxis for prevention of mother to child transmission of HIV, is mostly likely in accurate.  Please provide a reference like the current HIV management guidelines for Botswana.*

**Author’s response: **We have modified the manuscript with citations to the statement.

  

1. ***Reviewer’s comment*** *: The authors properly highlight the limitations of this study, some which have been highlighted above. However these are very strong limitations which will significantly reduce the impact of this paper. *

**Author’s response: **We agree with the reviewer that our study has some limitations that have been highlighted in the manuscript. We however think that this is important work on the use of the groundbreaking PANDAA  assay in ****an HIV-1 subtype C endemic setting. Future work will build on this project as more HDR mutation positions are covered by the assay.

 We appreciate your valuable comments and we will address all these in the revised manuscript.
